# Antimicrobial resistance in equine faecal Escherichia coli isolates from North West England

**DOI:** 10.1186/1476-0711-9-12

**Published:** 2010-04-07

**Authors:** Mohamed O Ahmed, Peter D Clegg, Nicola J Williams, Keith E Baptiste, Malcolm Bennett

**Affiliations:** 1 Department of Veterinary Microbiology and Parasitology, Faculty of Veterinary Medicine, AlFateh University, Tripoli, PO Box 13662, Libya; 2 Department of Comparative Molecular Medicine, School of Veterinary Science, University of Liverpool, Leahurst, Chester High Road, Neston, CH64 7TE, UK; 3 Department of Animal and Population Health, School of Veterinary Science, University of Liverpool, Leahurst, Chester High Road, Neston, CH64 7TE, UK; 4 Department of Large Animal Sciences, Faculty of Life Sciences, University of Copenhagen, Taastrup, 2630, Denmark

## Abstract

**Background:**

*Escherichia coli *isolates of equine faecal origin were investigated for antibiotic resistance, resistance genes and their ability to perform horizontal transfer.

**Methods:**

In total, 264 faecal samples were collected from 138 horses in hospital and community livery premises in northwest England, yielding 296 resistant *E. coli *isolates. Isolates were tested for susceptibility to antimicrobial drugs by disc diffusion and agar dilution methods in order to determine minimum inhibitory concentrations (MIC). PCR amplification was used to detect genes conferring resistance to: ampicillin (TEM and SHV beta-lactamase), chloramphenicol (*catI, catII, catIII *and *cml*), tetracycline *(tetA, tetB, tetC, tetD, tet E *and *tetG*), and trimethoprim (*dfrA1, dfrA9, dfrA12, dfrA13, dfr7*, and *dfr17*).

**Results:**

The proportion of antibiotic resistant isolates, and multidrug resistant isolates (MDR) was significantly higher in hospital samples compared to livery samples (MDR: 48% of hospital isolates; 12% of livery isolates, p < 0.001). Resistance to ciprofloxacin and florfenicol were identified mostly within the MDR phenotypes. Resistance genes included *dfr*, TEM beta-lactamase, *tet *and *cat*, conferring resistance to trimethoprim, ampicillin, tetracycline and chloramphenicol, respectively. Within each antimicrobial resistance group, these genes occurred at frequencies of 93% (260/279), 91%, 86.8% and 73.5%, respectively; with 115/296 (38.8%) found to be MDR isolates. Conjugation experiments were performed on selected isolates and MDR phenotypes were readily transferred.

**Conclusions:**

Our findings demonstrate that *E. coli *of equine faecal origin are commonly resistant to antibiotics used in human and veterinary medicine. Furthermore, our results suggest that most antibiotic resistance observed in equine *E. coli *is encoded by well-known and well-characterized resistant genes common to *E. coli *from man and domestic animals. These data support the ongoing concern about antimicrobial resistance, MDR, antimicrobial use in veterinary medicine and the zoonotic risk that horses could potentially pose to public health.

## Introduction

Bacterial resistance to antimicrobials is a global problem, and understanding the molecular basis of resistance acquisition and transmission can contribute to the development of new strategies to combat this phenomenon. Furthermore, a zoonotic component to bacterial antimicrobial resistance has been demonstrated [1-3]. Horses can be a reservoir of antibiotic resistant organisms and genetic determinants of resistance, that may affect veterinary treatment of animals, affect welfare and have economic implications. Such resistance can persist even without selective pressure [1]. Furthermore, antimicrobial use in animals can select for resistance genes that subsequently pose a risk to human public health through compromising the ability to treat infections [2-5]. The use of common antimicrobials in equine veterinary practices, the close contact between humans and horses, along with the risks and consequences such practices place on human health and therapy needs to be reassessed.

Commensal *E. coli *strains from humans and animals have been reported to express high resistance to common antimicrobial agents, harbouring antibiotic resistance genes such as *dfrA17 *and *dfrA12 *[6]. These resistance genes are commonly present on mobile genetic elements such as plasmids and integrons in clinical isolates of Gram-negative microorganisms [4]. Furthermore, resistance genes selected for in non-pathogenic bacteria may later transfer the acquired resistance to pathogenic bacterial species [7,8]. Thus, normal bacterial flora can play a key role as an acceptor and donor of antimicrobial resistance [9]. It has been suggested that in the UK, antibiotic resistance in *E. coli *of animal origin may arise by acquisition of resistance genes present in MDR bacteria in farm soil. Thus, these bacteria could play an important role in the dissemination of antimicrobial resistance genes to other soil microbes and gastrointestinal bacteria of grazing animals [10].

Whilst several studies in different animals including horses have analysed *E. coli *for their susceptibility to antimicrobial agents and genetic determinants [1,9,11,12], zoonotic components of antimicrobial resistance varies between countries [13] and studies of *Enterobacteriaceae *of equine origin in the UK are limited. To investigate factors that may influence zoonotic transmission of antibiotic resistant *E. coli *through faecal shedding by hospitalized and non-hospitalized horses we describe the prevalence and mechanisms of antibiotic resistance in *E. coli *isolated from equine faecal samples and analysed by susceptibility testing, PCR analysis and conjugation experiments.

## Methods

### Source of isolates

Faecal samples were collected over a six month period from an equine veterinary hospital and from two livery stables on the Wirral Peninsula, in north-west England. A total of 264 faecal samples were collected from 138 horses; 109 of these faecal samples (from 66 horses) came from the Philip Leverhulme Equine Hospital (PLEH) at the University of Liverpool and 155 faecal samples (from 72 horses) were randomly sampled at livery yards, comprising on average two faecal samples per horse. When scoring for the presence or absence of antibiotic resistant *E. coli*, faecal samples were taken as the unit of analysis. To investigate the antibiotic resistance profiles of *E. coli *shed in faecal samples, three *E. coli *colonies were isolated at random from each sample using semi-selective media (brilliant green broth and eosin methylene blue agar) and further confirmed biochemically using Api20E (bioMerieux) test.

### Antimicrobial Susceptibility testing

Antimicrobial susceptibility testing was performed using both disc diffusion and agar dilution methods, according to the British Society for Antimicrobial Chemotherapy BSAC guidelines.

### Disc diffusion method

Isolates were then subjected to disc diffusion testing according to the British Society for Antimicrobial Chemotherapy (BSAC) guidelines as described [14]. All faecal *E. coli *isolates resistant to at least one antimicrobial agent were stored at-80°C, until further analysis. Isolates were tested for resistance to the following antibiotics: ampicillin (30 μg) (and to cefotaxime (30 μg) and ceftazidime (30 μg) for extended resistance to cephalosporines and referred in this paper as potential ESBL producers for ampicillin resistant isolates), apramycin (30 μg), chloramphenicol (30 μg) (and to florfenicol (30 μg) for chloramphenicol resistant isolates), nalidixic acid (30 μg) (and ciprofloxacin(1 μg) for nalidixic acid resistant isolates), tetracycline (30 μg), trimethoprim (2.5 μg), streptomycin (10 μg), spectinomycin (25 μg), sulphonamides (100 μg) and gentamicin (10 μg). Stringent criteria were adopted for defining multidrug drug resistance (MDR), including resistance to at least four classes of antimicrobial agents. Extended resistance to cephalosporins was provisionally assigned following BSAC recommendations and these strains thereafter referred to as potential ESBL producers. To determine florfenicol resistance in chloramphenicol resistant isolates we adopted a criterion of R ≤ 18 mm and R ≤ 13 mm for apramycin resistance, following personal communication with J. M. Andrews, BSAC.

### Minimum inhibitory concentration (MIC) determination using agar dilution method

The MICs of resistant *E. coli *isolates were determined for each of the following antibiotics: ampicillin, chloramphenicol, ciprofloxacin, tetracycline and trimethoprim using the agar dilution method as described previously [15], and evaluated according to BSAC guidelines.

### Identification of antibiotic resistance genes

PCR amplification was used to identify genes responsible for resistance to: ampicillin, chloramphenicol, trimethoprim and tetracycline. In total, six different PCR protocols were applied and 18 antibiotic resistant genes were investigated. The method for DNA extraction was adapted from a method described by Kimata et al. [16]. Six PCR protocols were applied to detect specific genes according to the resistance phenotype, as follows: TEM and SHV β-lactamase genes for isolates exhibiting ampicillin resistance [17]; *catI, catII, catIII*, [18] and *cmlA *[19] for chloramphenicol resistance; *tetA, tetB, tetC, tetD, tetE *and *tetG *for six genes responsible for tetracycline resistance [20];*dfrA1, dfrA9 *[21] and *dfrA12, dfrA13, dfrA7, dfrA17 *[22] for trimethoprim resistance genes. PCR products of *dfrA7 *and *dfrA17, dfrA12 *and *dfrA13 *were cleaved using 20 U *EcoRV *and *pst1 *(Sigma) respectively. Positive controls were laboratory strains 7071 (*dfr12*) and 7082a (*dfr17*). Positive controls for the other resistance genes were DNA samples from bacterial isolates previously characterized and sequenced in-house. Table 1 lists all primer pairs used.

**Table 1:** Details of the primers used for PCR protocols.

*Primers sequences 5' to 3'*	*amplicon size (bp)*
***Shv***: CACTCAAGGATGTATTGTG, TTAGCGTTGCCAGTGC	885
***Tem***: TCGGGGAAATGTGCGCG, TGCTTAATCGTGAGGCACC	971
***CatI***: AGTTGCTCAATGTACCTATAACC, TTGTAATTCATTAAGCATTCTGCC	585
***CatII***: ACACTTTGCCCTTTATCGTC, TGAAAGCCATCACATACTGC	495
***CatIII***: TTCGCCGTGAGCATTTTG, TCGGATGAGTATGGGCAAC	508
***cmlA***: CCGCCACGGTGTTGTTGTTATC, CACCTTGCCTGCCCATCATTAG	698
***tet*B**: TTGGTTAGGGGCAAGTTTTG, GTAATGGGCCAATAACACCG	659
***tet*C**: CTTGAGAGCCTTCAACCCAG, ATGGTCCTCATCTACCTGCC	418
***tet*D**: AAACCATTACGGCATTCTGC, GACCGGATACACCATCCATC	787
***tet*A**: GCTACATCCTGCTTGCCTTC, CATAGATCGCCGTGAAGAGG	210
***tet*E**: AAACCACATCCTCCATACGC, AAATAGGCCACAACCGTCAG	278
***tetG***: GCTCGGTGGTATCTCTGCTC, AGCAACAGAATCGGGAACAC	210
***dfr1***: ACGGATCCTGGCTGTTGGTTGGACGC, CGGAATTCACCTTCCGGCTCGATGTC	254
***dfr9***: ATGAATTCCCGTGGCATGAACCAGAAGAT, ATGGATCCTTCAGTAATGGTCGGGACCTC	399
***dfrA7, dfrA17***: GTCGCCCTAAAACAAAGTTA, CGCCCATAGAGTCAAATGT	195
***dfr12, dfr13***: CCGTGGGTCGATGTTTGATG, GCATTGGGAAGAAGGCGTTCAC	485

Primer sequences and amplification protocols were taken from the following sources:: TEM & SHV β-lactamase genes [17];*catI, catII, catIII *[18]; *cmlA *[19];*tetA, tetB, tetC, tetD, tetE and tetG *[20];*dfrA1, dfrA9 *[21];*dfrA12, dfrA13, dfrA7, dfrA17 *[22]

### E. coli conjugation

Conjugation experiments were carried out on isolates susceptible to nalidixic acid and resistant to ampicillin (n = 35), using the nalidixic acid resistant *E. coli *K12 (developed using *E. coli *NTCC 10536) as a recipient strain. *E. coli *K12 was inoculated into 20 ml nutrient broths (LabM) and incubated overnight at 37°C. Resistant *E. coli *strains (donor strains) were inoculated into separate 3 ml nutrient broths and incubated overnight; 4 ml of recipient strain was then added to the donor strain and incubated at 37°C for one hour. Broths were then streaked onto agar plates containing nalidixic acid (30 μg/ml) plus ampicillin (8 μg/ml). Plates were incubated for 24 hours. Successful transconjugants were subcultured onto nutrient agar for susceptibility testing by disc diffusion as previously described. The resistance profiles of the transconjugants were compared to the resistance profile of the original strains. Gene profiles of the donor isolates, characterized by PCR, were described prior to the tranconjugation experiments

### Statistical analysis

Data was analysed with SPSS statistical software, using the z test for comparing two proportions.

## Results

Antibiotic resistant *E. coli *isolates were obtained from both sources of faeces, but with a significant difference in the prevalence of resistant isolates between the hospital and livery premises. For an overview of the frequency of antibiotic resistant faecal *E. coli*, comparison was made at the level of the faecal sample, the unit probably most closely associated with zoonotic transmission. Among samples from the hospitalized horses, 89/109 contained at least one antibiotic-resistant *E. coli *isolate, whereas only 38/155 of the livery-derived equine samples contained resistant isolates, (p < 0.001). A collection of 296 antibiotic resistant *E. coli *isolates, consisting of 219 and 77 resistant isolates from the hospital and livery premises respectively, were selected for further analysis, allowing comparison of resistance phenotypes (including MDR) in individual isolates (summarised in Table 2). The proportion of MDR resistant isolates, identified by disk diffusion, was significantly greater in the hospital-derived isolates (106/219; 48%) than in livery-derived isolates (9/77; 12%, p < 0.001). MIC testing confirmed these resistance phenotypes in >90% of the isolates.

**Table 2:** Resistance profiles and MDR frequency in *E. coli *isolates from equine faecal samples.

Abs	No. of faecal samples with at least one resistant *E. coli; *disc diffusion method (%)	No *E. coli *isolates exhibiting resistant phenotype	Proportion of isolates resistant by disc diffusion method, which were confirmed by MIC	No MDR-positive faecal samples (% faecal samples)
AMP	89 (33.7%)	191	93.0%	57 (21.6%)
APR	1 (0.4%)	1	-	1 (0.4%)
NAL	36 (13.6%)	72	-	35 (13.3%)
CIP	28 (10.6%)	65	93.8%	28 (10.6%)
CHL	49 (18.6%)	102	100%	47 (17.8%)
FLO	9 (3.4%)	14	-	9 (3.4%)
TET	92 (34.8%)	198	93.4%	57 (21.6%)
TRI	135 (51.0%)	279	95.0%	57 (21.6%)

Abbreviation: ABs, antimicrobials; AMP, ampicillin; APR, apramycin; NAL, nalidixic acid; CIP, ciprofloxacin; CHL, chloramphenicol; FLO, florfenicol; TET, tetracyclin; TRI, trimethoprim

### Antimicrobial resistance and multidrug resistant (MDR) E. coli

The number of isolates showing resistance to each antimicrobial agent were: trimethorpim (n = 279), tetracycline (n = 198), ampicillin (n = 191), potential ESBLs (n = 17), chloramphenicol (n = 102), florfenicol (n = 14), nalidixic acid (n = 72), ciprofloxacin (n = 65), apramycin (n = 1), aminoglycosides (n = 249), sulphonamides (n = 282) and gentamicin (n = 59) (Table 2). Results obtained through MIC testing were in good agreement (>93%) with those obtained by the disc diffusion test (Table 2).

Overall, 115 (38.8%) isolates demonstrated an MDR phenotype (resistance to four or more classes of antibiotics): 106 MDR isolates were of hospital origin and nine isolates were from livery premises. The resistance profiles of the MDR isolates fell mostly into three distinct groups: AMP, CHL, TET, TRI, NAL (n = 27/23.4%); AMP, CHLTET, TRI (n = 21/18.2%); AMP, TET, TRI, NAL (n = 9/7.8%).

### Antimicrobial resistance genes

In total, nine resistance genes were identified (Table 3). Trimethoprim resistance was attributable to *dfr *genes in 93% (260/279) at the following frequencies: *dfr1 *(40.3%), *dfr17 *(28%), *dfr12 *(17.3%) and *dfr9 *(0.3%). Of the tetracycline resistant isolates, 86.8% (172/198) were positive by PCR for *tet *genes, with *tetB *the most prevalent gene (71%), followed by *tetA *at 18% and *tet(A*+*B*) at 11%. Amplicons of the *catI *gene were obtained from 73.5% (75/102) of chloramphenicol resistant isolates, all of which were of hospital origin. Only one of these was also resistant to florfenicol. Of the ampicillin resistant isolates, the TEM β-lactamase genes was identified in 91% (174/191) and only one isolate was positive for the SHV β-lactamase genes.

**Table 3:** Summary of results showing the identification and distribution of antibiotic resistance genes in *E. coli *isolates from equine faecal samples taken from hospital and livery premises.

*Antibiotic(No. of resistant* *isolates investigated)*	*Source and number(N) of isolates*	*Identified by PCR*	*Not identified by* *PCR*	*Antibiotic resistance gene composition tested by PCR (details in table legend)*
AMP (191)	Hospital n = 177 Livery n = 14	169 5	8 9	**TEM & SHV β-lactamase genes**
CHL (102)	Hospital n = 97 Livery n = 5	75 0	22 5	***catI ****catII catIII cmlA*
TET (198)	Hospital n = 177 Livery n = 21	154 18	23 3	***tetB tetA ****tetC tetD tetE tetG*
TRI (279)	Hospital n = 209 Livery n = 70	195 65	14 5	***dfrA1 dfrA17 dfrA12 dfrA9 ****dfrA7, dfrA13*

All genes listed were tested by PCR amplification using gene-specific primer pairs listed in Table 1. Genes positively identified by PCR are shown in bold, and are listed according to their frequency of occurrence within each resistance group: AMP, **TEM lactamase genes 91%**, **SHV β-lactamase genes 0.6%**; CHL, ***catI *73.5%**; TET, ***tetB *71%, *tetA *18%**,***tetA+B *11%; **TRI, ***dfrA1 *40.3%**, ***dfrA17 *28%**, ***dfrA12 *17.3%**, ***dfrA9 *0.3%**. Those genes failed to give rise to detectable levels of PCR product are listed in normal type.

Abbreviations: AMP, ampicillin; CHL, chloramphenicol; TET, tetracycline; TRI, trimethoprim

### Conjugation experiments

Eight transconjugants (8/35) were obtained, and all were from hospital isolates (Table 4). The resistance profiles of the transconjugants were re-confirmed by disc diffusion testing and found to be identical to those of the donors. The resistance phenotype AMP, CHL, TET, TRI was the dominant (n = 6) followed by AMP, TET, TRI (n = 2).

**Table 4:** Resistant isolates that transferred resistance via conjugation, listed according to their resistance phenotypes.

Antibiogram	Donor genes contributing to the phenotype of each isolate
AMP, CHL, TET, TRI (n = 6)	*dfrA17, tetA, catI*,TEM β-lactamase genes
	*dfrA17, catI*,TEM β-lactamase genes
	*tetA, dfr1, dfrA17*,TEM β-lactamase genes
	*dfrA17, dfrA12, tetB*,TEM β-lactamase genes
	*dfrA12, tetA, tetB*,TEM β-lactamase genes, *catI*
	*dfrA1, dfrA1, tetB*,TEM β-lactamase genes, *catI*

AMP, TET, TRI (n = 2)	*dfrA17, dfrA12, tetB*,TEM β-lactamase genes
	*dfrA1, tetB*,TEM β-lactamase genes

Abbreviations: AMP, ampicillin; CHL, chloramphenicol; TET, tetracycline; TRI, trimethoprim; n = number of isolates

## Discussion

Our results show that hospitalized horses are more likely to shed antibiotic resistant *E. coli *strains, and their faeces are more likely to harbour MDR than those of horses in livery premises. The significantly higher prevalence of antibiotic resistance and of MDR found in hospitalized horses in this study fits well with previous observations [23]. All the horses at the PLEH are referrals from private veterinary practices, so many of the horses will have undergone treatment, which may include prior antibiotic administration. Thus, the higher prevalence of resistant *E. coli *in hospitalized horses could reflect conditions prior to arrival at the PLEH. Transportation and other stress factors have also been shown to increase the shedding of antibiotic resistant enteric bacteria [23], and this may also have influenced the prevalence of resistant *E. coli *strains in samples collected from the PLEH.

Ampicillin resistance in *E. coli *isolates described in this paper was largely associated with TEM β-lactamase genes, with only one isolate positive for SHV β-lactamase genes. This agrees with other reports that TEM β-lactamase genes (i.e. TEM-1 β-lactamase gene) are the most prevalent in ampicillin resistant *E. coli *of animal origin, as well as being commonly reported in human *E. coli *isolates of hospital origin [23]. Furthermore, this study identified 17 isolates as resistant to cephalosporins (i.e. potential ESBL producers), all of which were positive for TEM β-lactamase genes, with MICs for ampicillin ≥ 256 μg/ml (except for two isolates against, which the MICs = 128 μg/ml). The prevalence of extended spectrum beta-lactamase (ESBL) resistance in European countries of *E. coli *human isolates is reported to be around 3.9% [24] with variations between countries. ESBL-targeted drugs are being used more frequently, but may result in mutations of TEM and SHVβ-lactamase genes, as well as the widely prevalent *ctx-m *types [25]. For the potential ESBL producers more identification and confirmation is required and further genotypic analysis in needed. Our results showed that *E. coli *resistance genes from horses are similar to those found in other animals and humans, however these need further investigation, specifically by sequencing the TEMβ-lactamase PCR products.

In our study, the *tetB *gene was the most prevalent (71%) tetracycline resistance gene, followed by *tetA *(18%), and no other *tet *gene was identified. This prevalence pattern has also been reported in *E. coli *strains from various animals, including horses [26]. The *tetB *gene has the widest host range among gram-negative pathogens [27]. In gram-negative bacteria, *tetA *and *tetB *efflux genes are widely distributed and normally associated with plasmids, of which most are conjugative [27] and there is evidence for a correlation between the widespread distribution of tetracycline resistance genes and the sub-therapeutic antimicrobial use of tetracycline [28]. However, in the UK, tetracyclines are used relatively rarely in equine veterinary medicine, suggesting that the *tet *resistance genes identified here may have been acquired by co-selection mechanisms, as discussed below.

The *catI *gene, responsible for most of the plasmid-mediated resistance to chloramphenicol, was the only *cat *gene detected in the chloramphenicol resistant isolates. Most of chloramphenicol resistant isolates (47/102) were of the MDR phenotype (Table 2), suggesting that resistance to chloramphenicol is likely to be part of a multiple resistance system. The non-enzymatic chloramphenicol resistance gene (*cmlA*) also confers resistance to florfenicol. However, that *cmlA *gene was not identified by PCR, suggesting that the observed flofenicol resistance was possibly due to *flo *gene, although some chloramphenicol resistant genes can also be responsible for flofenicol resistance [29]. The use of chloramphenicol in UK veterinary medicine is generally restricted to topical application as a treatment for ophthalmic conditions, and is hardly ever used systemically. Chloramphenicol resistance was almost exclusively found in hospital-derived samples indicating that, as with tetracycline resistance, chloramphenicol resistance has most probably been co-selected via linked trimethoprim and ampicillin resistance genes.

A large proportion (93%) of the trimethoprim resistant isolates were positive for at least one of the *dfr *genes, that are commonly encoded on mobile genetic elements. Particularly, *dfrA1 *has spread rapidly on the transposon Tn7 to become the most prevalent gene responsible for trimethoprim resistance in the UK [30], and the most prevalent in our study, followed by *dfrA12 *and *dfrA17*. The *dfrA9 *was found only in one isolate in the present study. The *dfrA9 *gene, first reported in porcine *E. coli *strains, has also been reported in veterinary isolates and spread to human strains probably as a consequence of the extensive use of potentiated sulphonamide products (e.g. Sulfadiazine/trimethoprim) in veterinary medicine [31]. Reportedly, *dfr *genes are mostly conjugally transferable [6,32].

A previous study on horses has documented *in vitro *conjugal transfer of antibiotic resistant genes between genera of *Enterobacteriaceae *[12]. In studies on *E. coli *isolates of human origin, the wide dissemination of *dfrA17 *in urinary *E. coli *isolates is due mainly to the horizontal transfer of class 1 integrons, via conjugative plasmids [32]. Similarly, horizontal transfer through conjugative plasmids has been reported to be responsible for the wide dissemination of mobile genetic elements (e.g. class 1 integrons) in *E. coli *isolates from humans and animals [6]. Classes of integrons are widely reported in *Enterobacteriaceae *from animals [6,10] and in *E. coli *of animal origin, including horses [1]. Some of our isolates possess the ability to transfer resistance to a recipient (*E. coli *K12), see table 4, clearly indicating that the resistance is harboured on mobile genetic elements.

Our MIC determination and genetic analysis of resistant isolates suggests that in horses, antibiotic resistance is conferred by the same *E. coli *resistance genes found in other animal species. In total, 115/296 (38.8%) of our isolates showed MDR. Interestingly, MDR transferred in the conjugation studies, in which all transconjugants showed resistance to ampicillin and trimethoprim (table 4), indicating that both resistance profiles are encoded on mobile genetic elements. Antibiotic resistance can arise in the absence of selective pressures where antibiotic resistance genes are linked on a mobile genetic element; in these cases, exposure to a single antimicrobial agent has been shown to give rise to co-selection of multiple antibiotic resistance genes. Furthermore, stopping treatment, and consequent removal of selective pressure, does not necessarily lead to the loss of resistance [4,33]. Resistance to a range of antimicrobials can thus be selected for by administrating one, or a subset, of antimicrobials [9]. The shedding of resistant bacteria could thus produce a reservoir of resistant bacteria in the environment [4,10]. This type of mechanism may account for the presence of genes conferring resistance to tetracyclines and chloramphenicol, which are very rarely used therapeutically in equine veterinary medicine in the UK. Further work is required to define the multidrug resistant mechanisms, that may be responsible for the high level of prevalence of the resistance profiles (AMP, CHL, TET, TRI, NAL; AMP, CHL, TET, TRI; AMP, TET, TRI, NAL) we have identified. That most of these were from hospital sources might explain the possible role of antimicrobials in the dissemination and development of resistance in this environment.

## Conclusions

To our knowledge this is the first study of antibiotic resistant *E. coli *in equine faeces in the UK. Many of the genes we have identified as responsible for antibiotic resistance in equine *E. coli *are commonly found in other domestic animals and humans. Antibiotic resistance found in horses probably originates from, and is selected by, the same sources and mechanisms as in other animal species. Thus, in the UK, horses may be both recipients, and sources of the zoonotic transmission of antibiotic resistance and MDR, as well as providing an extensive reservoir for antimicrobial resistance genes.

## Competing interests

The authors declare no relationship (commercial or otherwise) that may constitute a dual or conflicting interest.

## Authors' contributions

MOA was responsible for study design, data collection and writing the manuscript. NJW provided strains and resistance data, and PDC organized samples collection within the hospital. All the authors contributed to the study design, the analysis of the results and writing this manuscript. All authors read and approved the final manuscript.
